# Exploring the impact of air conditioning on the indoor air quality of UK homes

**DOI:** 10.1088/1742-6596/3140/9/092012

**Published:** 2025-11-01

**Authors:** G Petrou, A Hamada, M Ashdown, C Simpson, M Davies

**Affiliations:** 1 UCL Institute for Environmental Design and Engineering, London, UK; 2 UCL Energy Institute, London, UK

## Abstract

Increasingly warm summers driven by climate change are resulting in the uptake of air conditioning (AC) in countries such as the United Kingdom (UK) where overheating has not traditionally been a concern. The split-type AC, one of the most popular types of AC, recirculates indoor air that has been cooled and filtered. Despite growing interest in indoor air quality, there is a scarcity of evidence on the overall impact of AC on the concentration of indoor air pollutants in UK homes. EnergyPlus and CONTAM were used to model the household average exposure to indoor- and outdoor-sourced fine particulate matter (PM_2.5_), and radon for an archetypical bungalow model with 14 combinations of AC usage and filter efficiencies: 1 scenario of only using natural ventilation (NV), 9 scenarios with only AC, and 3 mixed-mode scenarios. Compared to the NV scenario, AC-only and mixed-mode scenarios resulted in modest reductions (up to 4.5%) in PM_2.5_ exposure levels when particle filtration efficiency was 30%, but modest increase when filter efficiency was 10% (up to 5.8%). Annual mean radon exposure increased with the use of AC by 14.6-19.3%. Based on these findings, AC uptake could reduce the concentration of pollutants that can be filtered, even if they are in part generated indoors, if the filter efficiency is sufficiently high. For other indoor-sourced pollutants, the reduction in ventilation could increase their indoor concentrations with potentially detrimental effects for health.

## 1 Introduction

People in industrialised countries spend between 80% and 90% of their time indoors [1], with 66% within their own homes [2]. Personal exposure to air pollution in the indoor environment may thus be greater than outdoor exposure. Yet, indoor air quality (IAQ) remains understudied compared to outdoor air quality, despite high heterogeneity of pollutants compared to outdoors, with comparatively fewer IAQ guidelines and less regulatory oversight [3].

IAQ depends on the strength of indoor and outdoor sources of air pollution, as well as factors such the transportation, deposition and reaction of pollutants [3]. Climate change could impact IAQ in several ways [4], including through a change in ventilation levels and the adoption of cooling technologies in previously heating-dominated countries. There is some evidence that recirculating air conditioning (AC) units may have a detrimental effect on air quality compared to the use of natural ventilation. Cheung and Jim (2019) continuously monitored carbon monoxide (CO), carbon dioxide (CO_2_), particulate matter with aerodynamic diameter smaller than 10 μm (PM_10_) and 2.5 μm (PM_2.5_), and volatile organic compounds (VOCs) in 72 tiny homes in Hong Kong, each with an average floor space per occupant of 5.3 m^2^ [5]. Both CO and CO_2_ showed elevated concentrations while the air conditioning was in use, with mean CO_2_ levels ranging from 500 ppm to 1750 ppm. PM_10_, PM_2.5_, and VOCs increased during the initial stage of AC operation (immediately after being switched on) before stabilising. It was hypothesized that this was due to dust accumulating in the filters and evaporators.

On the other hand, Bell et al. (2009) have indicated (using a Bayesian hierarchical model on population level data) that communities with higher prevalence of AC use experienced smaller effects of outdoor PM_2.5_ concentration on hospitalisations related to cardiovascular issues [6], and Chuang et al. (2017) empirically demonstrated that long term AC filtration was associated with a reduction in harmful pollutants, as well as improvements to cardiovascular health in a study of 200 homemakers in Taipei [7].

Given this mixed picture, and a lack of evidence in the UK context, this paper aims to model the impact of recirculating AC operation on the indoor air quality of a UK bungalow home. Specifically, this study will focus on the effect of AC operation on PM_2.5_ and radon, two pollutants with established health impacts [8].

## 2 Methods

Indoor PM_2.5_, both from indoor and outdoor sources, and radon concentration was modelled in CONTAM-EnergyPlus for an archetypical, naturally ventilated, 1950s bungalow located in London, England. A detailed description of the archetype, including its thermophysical characteristics, and the CONTAM-EnergyPlus co-simulation approach is provided by Wang et al. [9]. The following sections summarise the modelling approach.

### 2.1 Building modelling

Modelling was carried out for a bungalow model following retrofit in accordance to Approved Document L 2021, the government approved guidance to demonstrate compliance with the Building Regulations [10]. Bathrooms and kitchens were equipped with intermittent extract fans, and the double-glazed windows had trickle ventilators installed, as detailed in Approved Document F [11]. The trickle ventilators were always open, while the intermittent extract fans were on during cooking (in the kitchen) and when the bathroom was in use. Two adults and two young children were assumed to live in the bungalow, with occupancy profiles described in previous work [12].

PM_2.5_ was assumed to be generated indoors during cooking (1.6 mg/min), penetrating from the outdoors (penetration factor of 1 with windows open and 0.8 otherwise [13]), and deposited at a rate of 0.39 h^-1^ [14]. Outdoor PM_2.5_ concentration was assumed to be 10 μg/m^3^, based on the UK target of annual mean PM_2.5_ concentrations of 10 μg m^-3^ or less by 2040 [15]. Radon is modelled as entering through the ground floor driven by temperature-dependent pressure differences between indoors and outdoors [9].

Simulations were run using a 5-minute timestep, a compromise between computational cost and data resolution, with the concentration (*c*) of each pollutant (*p*) reported separately for each room (*r*) at every timestep (*t*). The exposure for each occupant was estimated as follows:

(1) $$e_{p,i,t}=\Sigma_{r=1}^{R}o_{i,r,t}c_{p,r,t},$$

where *o*_*i*,*r*,*t*_ is an indicator variable that signifies whether individual *i* occupies room *r* for timestep *t*, taking values of 1 or 0. If present at home, each occupant is assumed to only occupy one room at each timestep, thus $\Sigma_{r=1}^{R}o_{i,r,t}=0$ *or* 1. By considering the total time steps in a given period (*T*) and the total number of occupants (*I*), the arithmetic mean of household exposure $\left({\bar{e}}_{p}^{a}\right)$ can be estimated:

(2) $${\bar{e}}_{p}^{a}=\frac{1}{I}\Sigma_{i=1}^{I}\frac{1}{T}\Sigma_{t=1}^{T}e_{p,i,t},$$

Equation 2 was used to estimate the household average exposure during a one-year period (1^st^ Jan to 31^st^ Dec), the summer (1^st^ Apr to 31^st^ Sept) and winter (1^st^ Jan to 31^st^ Mar and 1^st^ Oct to 31^st^ Dec).

A Design Summer Year (DSY) weather file, developed by the Chartered Institution of Building Services Engineers and representing a global warming level of 2 °C, was used.

### 2.2 Window Opening and Air-Conditioning Modelling

The study explored multiple AC and natural ventilation (NV) scenarios, shown in Table 1. In NV scenarios, the window opening availability in a zone was subject to the following conditions:

- It is the summer season (1^st^ April to 30^th^ of September) and the zone is occupied.
- The indoor operative temperature is higher than 22 °C.
- The outdoor air temperature is lower than the indoor air temperature.

**Figure 1:**
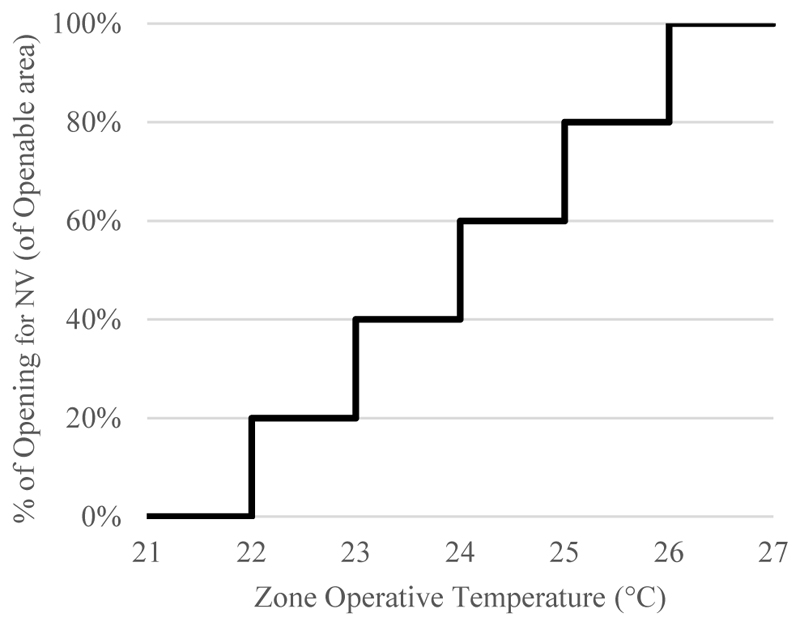
Percentage of opening increments in relation to zone operative temperatures.

An EnergyPlus Energy Management System (EMS) script was used to check these conditions at every timestep to control NV airflow in CONTAM. The operative temperatures at which windows would start to open (22 °C) and be fully open (26 °C) was based on Approved Document O [16], England’s government approved guidance to comply with Part O of the Building Regulations 2010. The percentage of the window opening was designed to increase incrementally from 0% to 100% of the openable area as the indoor zone operative temperature increases from 22 °C to 26 °C, as indicated in Figure 1. Given the relatively large window area (around 2.2 m^2^), it was assumed that half of the window was a fixed glass panel, meaning that 100% of the openable area, indicated in Figure 1, was equivalent to 50% of the total window area.

AC was modelled in CONTAM as a recirculation terminal (zonal) system that cools and filters the air, with no outdoor fresh air provided through the AC equipment. Cooling availability in a zone was subject to the following conditions:

- It is the summer season (1^st^ April to 30^th^ of September) and the zone is occupied.
- The indoor air temperature is higher than the cooling setpoint of 22 °C, 23 °C, or 24 °C.

Availability conditions for AC operation in each zone were checked at every timestep via an EnergyPlus EMS script that controls the cooling airflows in CONTAM.

In hybrid (mixed-mode) scenarios, where both NV and AC are available, windows were designed to be open when the operative temperature exceeds 22 °C according to NV availability conditions, but would be shut if cooling is switched ON – when the air temperature exceeds 24 °C.

Three PM_2.5_ filtration efficiencies in the AC recirculation air loop were assumed: 10%, 20%, and 30%. This was based on assuming a G4-graded filter is installed, with 20% efficiency assumed according to EN 779 (CEN 2012) [17], and ± 10% assumed as sensitivity analysis. The filters were assumed to have no effect on indoor radon concentration.

## 3 Results

### 3.1 PM_2.5_

**Figure 2:**
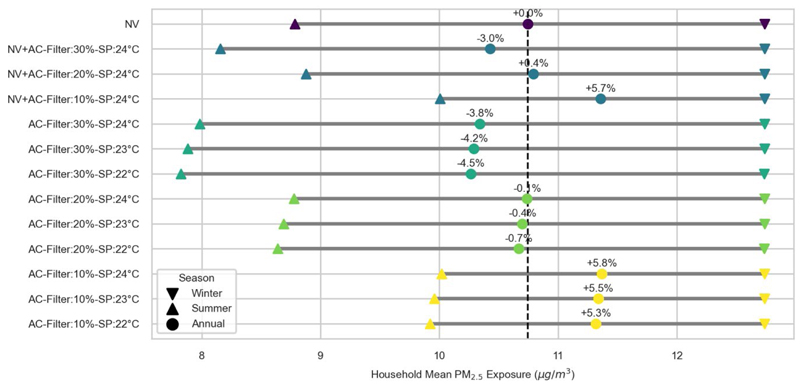
Household mean PM_2.5_ exposure for each scenario, averaged over the summer, winter and annual periods. Percentage differences are shown for the annual estimates, relative to the Natural Ventilation (NV) scenario (vertical dashed line).

Figure 2 summarises the summer, winter and annual mean household exposure to PM_2.5_ for each scenario. The Natural Ventilation (NV) scenario results in an annual mean household exposure of 10.7 μg/m^3^. Compared to the NV scenario, the use of AC particle filtration efficiency of 10% results in an increase in the annual mean household exposure of 5.3-5.8%, depending on the cooling setpoint. Assuming particle filtration efficiency of 20% results in annual mean household exposures that are comparable to the Natural Ventilation scenario. With particle filtration efficiency of 30%, the annual mean household exposure is 3.8-4.5% lower than in the case of NV. Where mixed-mode is employed, assuming particle filtration efficiency of 10% results in annual mean household exposure of 11.4 μg/m^3^, 5.7% higher than the NV scenario. Increasing the efficiency of the filters to 30% resulted in an annual mean household exposure of 10.4 μg/m^3^, approximately 3.0% lower case with only natural ventilation.

As expected, the winter mean household exposure remains unaffected by the use of air conditioning. The differences in annual mean household exposure are driven by differences in the summer period. Focusing on the summer average estimates, household exposure varies from 7.8 μg/m^3^ (AC Filter 30% - setpoint 22 °C) to 10.0 μg/m^3^ (AC Filter 10% - setpoint 24 °C).

### 3.2 Radon

**Figure 3:**
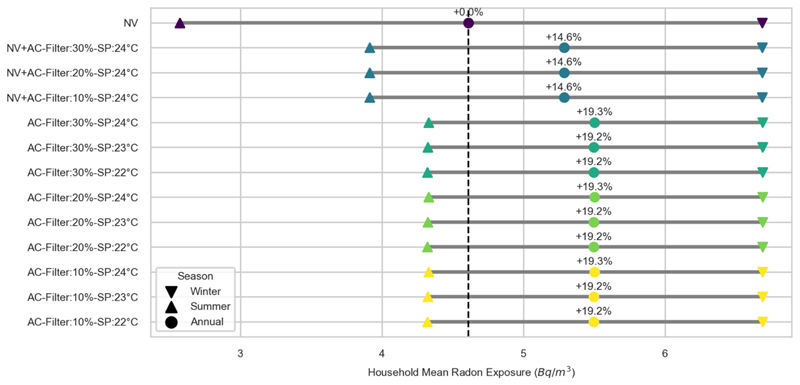
Household mean radon exposure for each scenario, averaged over the summer, winter and annual periods. Percentage differences are shown for the annual estimates, relative to the Natural Ventilation (NV) scenario (vertical dashed line).

As with PM_2.5_, the approach to summertime cooling does not affect the winter mean household radon exposure (Figure 3). For the summer and annual periods, the use of AC results in an increase in indoor radon concentration for all scenarios when compared to the NV scenario. The increase in annual mean household exposure ranges from 14.6% for the mixed-mode scenarios to 19.2-19.3% when only AC is in use. The impact of cooling setpoint is small, resulting in variation of 0.1%.

## 4 Discussion & Conclusions

The use of AC was shown to impact summer and annual average household modelled PM_2.5_ and radon exposure levels. The adoption of AC offered modest reductions of PM_2.5_ exposure (up to 4.5%) when particle filtration efficiency was 30%. However, with particle filtration efficiency of 10%, the PM_2.5_ exposure levels increased by up to 5.8% compared to a natural-ventilation only scenario. The choice of cooling setpoint had limited impact on exposure. This finding demonstrates the importance of filter efficiency in determining whether AC usage will have a beneficial or detrimental effect on indoor particle concentration. This finding will depend on the balance of indoor versus outdoor pollution sources, as well as the condition of filters and potentially their position in the room. It is worth noting that the annual exposure levels for all scenarios of this case study exceeded the air quality guideline level of 5.0 μg/m^3^ suggested by the World Health Organisation [18].

Another important finding relates to radon, where annual exposure levels were higher (by 14.6-19.3%) when AC was used for all scenarios. This was due to the reduction in ventilation level, leading to an accumulation of radon. While the levels of radon exposure were modest, well below the action level of 200 Bq/m^3^ set by the UK Health Security Agency [19], this was the result of assuming the bungalow model to be in London where soil radon levels are low. If comparable relative increases are observed in homes located in regions with high soil radon levels, the impact of AC could be substantial.

A key limitation of this work is the focus on a single case study. Factors such as dwelling typology, the strength of PM_2.5_ and radon sources, the climate scenario and occupancy assumptions can all contribute to the modelled concentrations. In addition, this work focused on long-term average concentrations and thus did not investigate changes to short-term pollutant exposure from AC operation. Nevertheless, this work provides insights on the potential impact of AC use on IAQ. Future work will expand this analysis by exploring this impact on to multiple dwelling types and for different strengths of pollutant sources, occupant and equipment assumptions. Further, planned research will explore the potential tradeoffs in IAQ from the adoption of AC with changes in heat exposure, energy use and costs.

## Figures and Tables

**Table 1:** The ventilation and air conditioning scenarios examined in the study.

Scenario configuration	Number of models
NV (@ 22 °C), no cooling	1
AC (@ 22, 23, 24 °C) x 3 PM_2.5_ filtration scenarios (10, 20, 30%)	9
NV (@ 22 °C) + AC (@ 24 °C) x 3 PM_2.5 _filtration scenarios (10, 20, 30%)	3
**Total number of scenarios modelled**	**14**
